# Stress-Induced Endocrine and Immune Dysfunctions in Caregivers of People with Eating Disorders

**DOI:** 10.3390/ijerph14121560

**Published:** 2017-12-13

**Authors:** Ángel Romero-Martínez, Luis Moya-Albiol

**Affiliations:** Psychobiology Department, University of Valencia, 46010 Valencia, Spain; luis.moya@uv.es

**Keywords:** caregivers, chronic stress, cortisol, eating disorders, immunoglobulin

## Abstract

Caregivers have to cope repeatedly with acute stressors in their daily lives, and this is associated with disturbances in the hypothalamic–pituitary–adrenal (HPA) axis and the immune system. Such disturbances could contribute to the development of health problems in informal caregivers of people with chronic illnesses, such as eating disorders (EDs). The main objective of this study was to examine endocrine (salivary cortisol levels (Csal)), immune (immunoglobulin-A (IgA)), and psychological (anxiety, mood, and anger feelings) responses to an acute psychological stressor in a sample of informal caregivers of individuals with EDs compared to a sample of non-caregivers. In addition, it also aimed to analyze the potential relationship of the aforementioned endocrine and immune response parameters with psychological variables in the caregivers. Caregivers had lower Csal and IgA levels at all assessment points except baseline. Moreover, they also exhibited lower Csal and IgA responses and greater worsening of mood in response to acute psychosocial stress than the non-caregivers, which suggests that caregivers had dampened endocrine and immune reactivity to acute stress. On the other hand, endocrine and immune parameters were unrelated to psychological variables. These findings advance our understanding of how a chronically stressed population reacts to acute stress, and should be considered for the development of effective interventions focused on stress management that could help caregivers to reduce their stress levels, which, in turn, would improve their health.

## 1. Introduction

Caregivers have to cope repeatedly with acute stressors in their daily lives, and this is associated with disturbances in the hypothalamic–pituitary–adrenal (HPA) axis and in the immune system. As these mechanisms regulate the body's response to stress, such disturbances could weaken caregivers’ health [1,2,3,4,5].

Previous studies in this field of research have based their conclusions on elderly caregivers of people with neurodegenerative pathologies, analyzing caregivers’ endocrine and/or immune response to an acute laboratory stressor [1,4,5] or studying their immune response after vaccination with a pneumococcal bacteria vaccine [3]. On the other hand, it has been demonstrated that caregivers’ age plays a role in their endocrine and immune response to stress, with elderly caregivers presenting HPA hyporeactivity and poor immune efficacy in comparison with younger caregivers and non-caregivers [6,7,8]. In elderly caregivers, however, the effects of age on psychobiological responses to acute stress overlap with those of caregiving, making it more difficult to discern differences between groups. Thus, it would be interesting to check whether the psychobiological disturbances observed in elderly caregivers are also present in younger caregivers.

A previous study compared the endocrine (measured through salivary cortisol levels), mucosal immunity (measured through salivary immunoglobulin-A (IgA) levels), and psychological (measured by assessing anxiety, mood, and anger feelings) responses to a standardized psychosocial acute stressor (a set of cognitive tasks in front of a four-member panel, with simulated video recording) in a group of male and female middle-aged caregivers of offspring with autism spectrum disorder (ASD) and a group of non-caregivers (also of both genders). It was found that caregivers had lower salivary cortisol (Csal) levels during the preparatory and stress periods than the non-caregivers. They also had lower IgA levels than non-caregivers during and after the stressor. Although no differences were found between groups in the magnitude of the Csal response, caregivers had a lower magnitude of salivary IgA response than non-caregivers. Regarding psychological response, caregivers presented a greater worsening of mood in response to acute stress than non-caregivers. Hence, the authors concluded that caregivers of offspring with ASD had dysregulated hormonal and immune responses to acute stress in comparison with non-caregivers [2]. The combination of caregiving and acute experimental stress might explain these endocrine, immunological, and psychological responses, as hypothesized by the cumulative stress hypothesis [9]. Although it has been reported that caregivers of people with eating disorders (EDs) have psychobiological disruptions associated with caring, there is a gap in the scientific literature regarding their endocrine and mucosal immunity response to acute laboratory stress.

With all this in mind, the present study examined the effect of a previously standardized psychosocial stressor [2] on psychobiological responses in long-term caregivers of people with EDs. To our knowledge, the present study is a pioneer in examining these responses to acute stress in a sample of caregivers of people with EDs. We hypothesized that these caregivers would have lower Csal and IgA levels and response to acute stress, as well as higher worsening of mood in response to acute stress than non-caregivers [2]. The second aim of the study was to examine the relationship between endocrine, immune, and psychological responses to acute psychosocial stress. As previous studies have revealed a powerful impact of negative affect on the HPA response to psychosocial laboratory stressors in a healthy population [10], we expected that a worsening of mood and an increase in anxiety and anger feelings would be related to an intense psychobiological response to acute stress. The analysis of these variables could help to offer a wider explanation of the relationship between chronic stress and health in caregivers. Further, such knowledge is relevant in the context of therapeutic and preventive programs to help us to detect high-risk individuals in a population that could be chronically stressed, and to apply effective interventions.

## 2. Method

### Participants

The final sample was comprised of 65 participants: 29 caregivers (10 men and 19 women) of offspring with EDs, and 36 non-caregivers (16 men and 20 women) who only cared for typically developing offspring. Parents of individuals with EDs were recruited from a hospital day care center specialized in EDs (Valencia, Spain). All parents participated voluntarily in the study and gave written informed consent in accordance with ethical principles regarding human research (Declaration of Helsinki). The study was approved by the University of Valencia’s Ethics Committee (code: H1360051962905).

We advertized in Valencia for male and female volunteers for the control group, establishing contact by email before screening applicants in interviews. An adequate control group was selected, comprising non-adolescent fathers and mothers of healthy offspring without any chronic illnesses or dependence due to disability. Additionally, parents in the control group had not been caregivers for anyone with a chronic illness in the previous two years. 

The ages and number of offspring in the experimental and control groups were similar. Anthropometric and demographic characteristics of ED parents and controls are summarized in Table 1.

## 3. Procedure

Each subject participated in one session in the psychobiology laboratories of the University of Valencia. Firstly, it was explained that they would be asked to provide saliva samples for hormone and immune marker measurements and to perform several behavioral tasks. Participants who were included in the study were instructed to abstain from eating, drinking stimulants (such as tea, coffee, or alcohol), brushing their teeth, and smoking during the 2 h period before arriving at the laboratory [2].

The experimental procedure was performed between 4:00 p.m. and 7:00 p.m., in order to minimize hormonal variations attributable to the circadian rhythm. Each session lasted approximately 2.5 h. After the participants arrived, data were gathered on anthropometric characteristics (age, weight, height). An initial pre-stress saliva sample was collected for measuring cortisol (Csal-1), and the participants were taken to the stress room. This room was sound-attenuated and temperature-controlled (21 ± 2 °C), with constant light levels during all sessions. After a habituation period, two more saliva samples were collected for measuring Csal and IgA (Csal-2 and IgA-1, respectively), and immediately afterwards, participants completed psychological questionnaires for the evaluation of pre-stress anxiety, anger, and mood. Once the questionnaires had been completed, general information regarding the stress stimuli was provided to the participants, who then remained silent for 3 min, and subsequently saliva samples were collected (Csal-3 and IgA-2).

Information regarding the evaluation of their performance during the stressor was then provided to the participants, and, after that, participants remained silent for 8 min (preparatory period). After this preparatory period, participants were exposed to a psychosocial stressor consisting of a 20-min session in front of a panel of two men and two women performing a set of cognitive tasks (memory test, Stroop test, mirror-drawing test, and arithmetic tasks). During this stressor period, a video camera was switched on to heighten the evaluative threat by simulating a recording, as suggested by other authors [11]. This stressor was an adapted version of the Trier Social Stress Task (TSST), a standardized protocol for the induction of moderate psychosocial stress in laboratory settings [12]. 

Between the second and third tasks of the stressor, the next saliva samples were collected (Csal-4 and IgA-3). Immediately after completing the stressors, further saliva samples were collected (Csal-5 and IgA-4), and then participants completed questionnaires for the evaluation of post-stress anxiety, anger, and mood. To assess the intensity of the stress experienced, the researchers ask the participants to appraise the task, in terms of the degree of perceived stress, to assess the intensity of the stress experienced and feelings of satisfaction regarding their performance. Afterwards, the participants were taken back to the first room where additional salivary samples for Csal measurements were collected at 10, 20, 30, 45, and 60 min after the stressor was completed (Csal-6, Csal-7, Csal-8, Csal-9, and Csal-10, respectively), and salivary samples for IgA were collected 10 and 20 min after the stressor (IgA-5 and IgA-6, respectively).

Finally, the participants completed an extended interview regarding other individual characteristics. In the case of caregivers, the researchers conducted an interview regarding the characteristics of the care recipient (diagnosis, gender, age, global activity, and dependence rating) and the status of the caregiver (years since the definitive diagnosis, time spent caregiving per week, burden, and level of worry regarding the future and the disorder).

### 3.1. Appraisal Scores

The task and the outcomes obtained were assessed with ad hoc questions rated on a 10-point scale. Participants were asked about the stress that the task caused (“On a scale from 0 (no stress) to 10 (extreme stress), how much stress did you experience during the task?”). They also answered a series of questions related to satisfaction with the outcome (“On a scale from 0 (not at all) to 10 (highly), how satisfied are you with the outcome obtained in the task?”) and to their attribution for the outcome (internal and external locus of control) (“On a scale from 0 (not at all) to 10 (highly), how dependent do you feel the outcome of the task was on you, your cognitive abilities and your intelligence?”, and “On a scale of 0 (not at all) to 10 (highly), how dependent do you feel the outcome of the task was on external factors, the events that occurred during the session, and the type of task?”). 

### 3.2. Psychological Response to Task

State anxiety was assessed using a Spanish-language culturally-adapted version of the “State-Trait Anxiety Inventory” (STAI-S) [13], which contains 20 items, ranked on a 4-point Likert scale. The reliability coefficient was 0.62.

A Spanish-language version of the “State-Trait Anger Expression Inventory-2” (STAXI-2) [14] was employed for measuring state anger. It contains 15 items ranked on a 4-point Likert scale and distributed into three subscales: Feelings, verbal, and physical expression. To reduce the number of tests, increase power for effect size, and aid interpretation within a conceptual framework, state anger subscales were combined into the single variable (S-Ang). Cronbach’s alpha ranged from 0.67 to 0.89.

Depressive mood states were measured using an abbreviated version of the profile of mood states (POMS) culturally adapted and validated in a Spanish population [15]. This questionnaire is composed of 29 items, rated on a 5-point Likert-type scale, grouped into five subscales (tension, depression, anger, vigor, and fatigue), with a Cronbach’s alpha higher than 0.80. In this study, only the depression subscale was employed.

### 3.3. Cortisol and IgA Analysis

The cortisol samples were collected with a Salivette cotton dental roll (Sarstedt, Rommersdolf, Germany) and were frozen at −20 °C immediately after collection and subsequently analyzed by radioimmunoassay. All samples from an individual were analyzed in duplicate in the same assay, and the values were averaged. The criterion for measurement replication was an inter-duplicate coefficient of variation of no more than 8%, and the intra- and inter-assay variation coefficients were 4.3% and 5.2%, respectively. The commercial Coat-a-Count Cortisol (DPC-Siemens Medical Solutions Diagnostics, Bad Nauheim, Germany) kit used employs a rabbit polyclonal antibody, and has a sensitivity of 1.4 nmol/L. 

The saliva samples for IgA were also collected with a Salivette cotton dental roll (Sarstedt, Rommersdolf, Germany). The samples were frozen at −20 °C immediately after collection until analysis in the laboratory by nephelometry using a BN-II system (Siemens, Munich, Germany) and N antiserum to human IgA produced in rabbit (Code no. OSAR-15, Dade Behring^®^, Marburg, Germany). While the coefficient of variation considered necessary for replication was fixed at 10%, the intra- and inter-assay variation coefficients obtained were 3.3% and 3.7%, respectively. The sensitivity was 0.2 mg/dL.

### 3.4. Data Analysis

After assessing the normality of the data using the Shapiro-Wilk test (*p* < 0.05), non-normal data were log_10_ transformed (Csal and IgA). Univariate analyses of variance (ANOVAs) were performed with “group” (caregivers and non-caregivers) and “gender” as the between-subject factors for anthropometric data (age and body mass index (BMI)) and baseline Csal and IgA stratifying by gender. Chi-square statistics were calculated for analyzing the frequencies of the demographic variables.

Csal and IgA changes in response to cognitive tasks were examined by repeated-measures ANOVAs. Further, to explore ‘‘group’’ and “gender” effects, repeated-measures analyses of covariance (ANCOVAs) were performed with “time” as the within-subject factor, “group” and “gender” as the between-subject factors, and selected variables which differ between groups as covariates. Greenhouse-Geisser corrections for degrees of freedom were applied where appropriate. For significant results, partial eta-squared was reported as a measure of effect size (η_p_^2^).

For psychological states, ANOVAs for repeated measures (pre- and post-stressor) were used with “period” as the within-subject factor and “group” and “gender” as the between-subject factors. Moreover, to explore ‘‘group’’ effects, repeated-measures ANCOVAs were performed with “time” (pre- and post-stressor) as the within-subject factor, “group” as the between-subject factor, and selected variables which differ between groups as covariates. Change scores in psychological responses were obtained as the difference between post-stressor and pre-stressor scores.

The magnitude of the endocrine and immune responses was estimated by calculating the area under the curve with respect to the increase (AUCi), using formulae derived from the trapezoidal rule, as previously described [16]. These formulae are simple additions of triangular and rectangular areas. Spearman’s or Pearson’s correlation coefficients were calculated to assess relationships between variables when appropriate for each group (caregivers and non-caregivers).

Data analyses were carried out using IBM SPSS Statistics for Windows, Version 22.0 (Armonk, NY, USA). *p* values < 0.05 were considered statistically significant. Average values are reported in the table as mean ± SD.

## 4. Results

### 4.1. Participant Characteristics

Caregivers did not differ from non-caregivers in BMI, socio-demographic characteristics, or baseline Csal and IgA levels. In contrast, differences were observed in age and menstrual status, F(1, 64) = 60.87, *p* < 0.001, η_p_^2^ = 0.50, and *χ*^2^ (3) = 11.24, *p* = 0.01, with caregivers being older and more likely to be in menopause than non-caregivers. For this reason, these variables were included as covariates in the subsequent analysis. 

### 4.2. Appraisal Scores

Caregivers obtained similar appraisal scores associated with the tasks to controls for perceived stress (5.17 ± 2.62 and 4.97 ± 1.72, respectively), satisfaction (4.86 ± 2.18 and 5.33 ± 1.15, respectively), and internal (6.48 ± 2.67 and 6.53 ± 1.98, respectively) and external (3.79 ± 2.86 and 3.36 ± 2.04, respectively) control.

### 4.3. Endocrine and Immune Responses to Acute Stress

The psychosocial stressor employed in this study was found to be effective, as indicated by the significant ‘‘time’’ effect on Csal in the total sample, ε = 0.26; F(2.37, 149.11) = 9.53, *p* < 0.001, η_p_^2^ = 0.13. When analyzing each group separately, a significant ‘‘time’’ effect was found in both caregivers, ε = 0.23, F(2.03, 56.87) = 2.08, *p* = 0.03, η_p_^2^ = 0.07, and non-caregivers, ε = 0.27, F(2.43, 82.64) = 7.93, *p* < 0.001, η_p_^2^ = 0.19. In caregivers, Csal decreased from baseline to the preparatory period and from then to the tasks, subsequently decreasing further until the recovery period (*p* < 0.05 for all comparisons). In non-caregivers, Csal followed a similar pattern to that observed in the caregivers (*p* < 0.05 for all comparisons).

**Figure 1:**
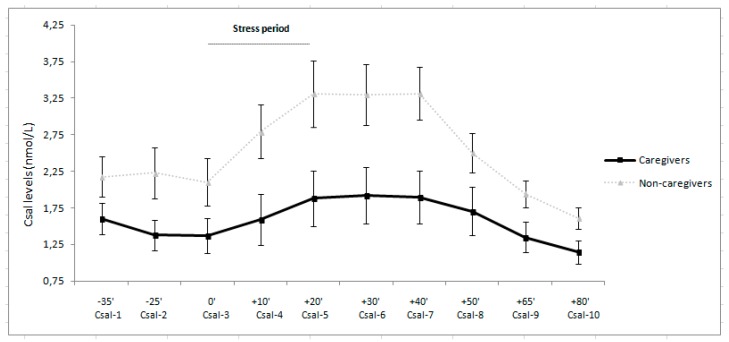
Estimated marginal means and standard errors of means of salivary cortisol (Csal) levels in caregivers and controls.

In the case of Csal, a significant ‘‘group’’ effect was found, F(1, 56) = 5.55, *p* = 0.022, η_p_^2^ = 0.09, with caregivers having lower Csal levels than non-caregivers at all time points assessed except baseline (*p* < 0.05) (see Figure 1). Concerning the magnitude of the response, a significant ‘‘group’’ effect was also found in AUCi for Csal, F(1, 63) = 3.74, *p* = 0.05, η_p_^2^ = 0.06, with caregivers having a lower Csal response to stress than non-caregivers.

Regarding IgA, a significant “time” effect was found in the total sample, ε = 0.80, F(4.05, 254.91) = 5.15, *p* < 0.001, η_p_^2^ = 0.08. When analyzing each group separately, a significant “time” effect was found in both caregivers, ε = 0.74, F(3.69, 99.67) = 2.27, *p* = 0.05, η_p_^2^ = 0.05 and non-caregivers, ε = 0.80, F(4.05, 254.91) = 5.15, *p* < 0.001, η_p_^2^ = 0.08. In caregivers, IgA levels decreased from baseline to the preparatory period, then increased to the tasks, and finally, decreased to the recovery period (for all *p* < 0.05). The IgA levels followed a similar pattern in non-caregivers (for all *p* < 0.05).

**Figure 2:**
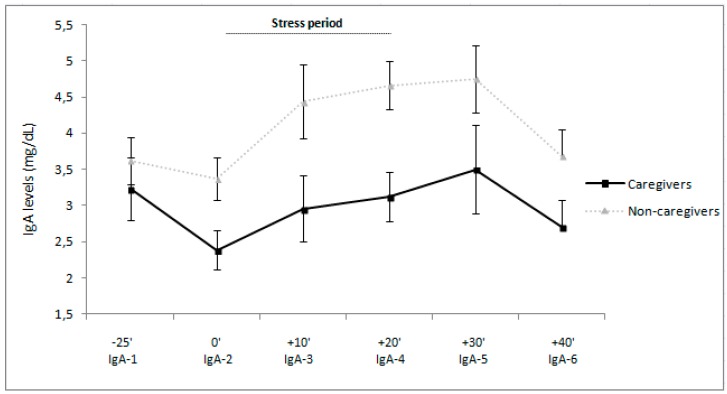
Estimated marginal means and standard errors of means of immunoglobulin-A (IgA) levels in caregivers and controls.

Further, a main effect of “group” was found, F(1, 58) = 8.09, *p* = 0.12, η_p_^2^ = 0.12, caregivers showing lower IgA levels than non-caregivers (see Figure 2). Concerning the magnitude of the response, a significant “group” effect was found also in AUCi for IgA, F(1, 64) = 3.96, *p* = 0.05, η_p_^2^ = 0.06, with caregivers having lower IgA response to stress than non-caregivers. 

### 4.4. Psychological Responses to Acute Stress

For anxiety, a significant effect of “time” was found, F(1, 64) = 34.27, *p* < 0.001, η_p_^2^ = 0.35. When analyzing each group separately, a significant “time” effect was found in both caregivers, F(1, 28) = 14.20, *p* = 0.001, η_p_^2^ = 0.34 and non-caregivers, F(1, 35) = 19.83, *p* < 0.001, η_p_^2^ = 0.36. Both groups increased their states of anxiety after the tasks. 

When analyzing anger, a significant effect was found for “time” in the S-Ang for all the sample, F(1, 64) = 5.45, *p* = 0.023, η_p_^2^ = 0.08. When analyzing each group separately, a significant “time” effect was found in both caregivers, F(1, 28) = 2.66, *p* = 0.05, η_p_^2^ = 0.09 and non-caregivers, F(1, 35) = 2.79, *p* = 0.05, η_p_^2^ = 0.07. Both groups increased their states of anger after the tasks.

Nonetheless, there were no significant “group” or “time × group” interactions for anxiety state and/or S-Ang, or in the magnitude of these responses (for anxiety 7.14 ± 10.20 and 6.67 ± 8.98, respectively; for S-Ang 0.34 ± 1.14 and 0.23 ± 0.83, respectively).

The laboratory stressor was not shown to be efficient for eliciting alterations in depressive mood for the total sample, since the factor “time” was not significant in the POMS depression score. When analyzing each group separately, a significant “time” effect was found in non-caregivers, F(1, 35) = 3.42, *p* = 0.05, η_p_^2^ = 0.09. There were no significant changes in POMS depression scores in caregivers, but non-caregivers experienced worse moods after the task. A significant “group” effect was found for POMS depression score, F(1, 63)= 4.58, *p* = 0.037, η_p_^2^ = 0.07, with caregivers obtaining higher depression scores than non-caregivers. Furthermore, there was a significant difference between groups in the change in POMS depression scores, with smaller changes in scores among caregivers than non-caregivers (−0.27 ± 3.91 and 0.64 ± 2.07, respectively).

### 4.5. Relationships of Baseline CSAL and AUCi for Csal and IgA with Baseline Psychological and Change Scores

Pearson’s and/or Spearman’s correlation analysis did not indicate any significant relationships between baseline or AUC_i_ Csal and IgA with baseline or changes scores in psychological variables in caregivers or non-caregivers.

## 5. Discussion

As previously found in caregivers of people with ASD [2], caregivers of people with EDs, specifically anorexia and bulimia nervosa, have lower Csal and IgA levels than non-caregivers during all periods except at baseline. Moreover, they also had lower Csal and IgA responses in response to acute psychosocial stress than the non-caregivers. Hence, we can conclude that caregivers had dysregulated stress-induced hormonal and immune responses compared to non-caregivers. On the other hand, these endocrine and immune parameters were unrelated to psychological variables in both groups.

Previous studies in the field have not reported significant differences in Csal levels when comparing elderly caregivers of people with dementia and non-caregivers [1,14,15]. Thus, HPA hyporeactivity to stress and hypocortisolism could be characteristic of caregivers of people with non-degenerative conditions. This could be explained by the fact that caregivers of people with EDs or neurodevelopmental disorders such as ASD worry about the future of their offspring when the parent cannot provide care [17]. Hence, these concerns about the future may add pressure to caregiving commitment. The above-described endocrine profile could be attributed to stress habituation, and this would support the hypothesis that caregivers have a blunted psychobiological response to stress [2].

Chronic stress results in a dysregulation of immune activity, which implies a non-adaptive mechanism [17,18,19,20,21,22]. In the present study, caregivers had lower levels of IgA and a weaker immune response than non-caregivers. As low levels of this immunoglobulin imply higher risk of respiratory and gastrointestinal infections [22], this could increase caregivers’ vulnerability to develop this kind of diseases and a slow disease recovery. Indeed, caregivers may be showing a non-adaptive profile of response, as observed in chronically stressed populations, in that they lack the immune competence that would be expected in acute events. This is congruent with two previous studies in caregivers which concluded that caregivers tend to present a dampened immune response to acute stress [1,2]. It remains unclear, however, whether the dampened responses of caregivers when coping with acute stress are associated with an adaptive redistribution of immune resources in acute stress, or a stable disruption in immune competence.

Endocrine and immune responses were not found to be associated with psychological variables in caregivers, suggesting that acute mood changes are not involved in the dampened Csal and suppressed IgA response to acute stress. Notably, de Andrés-García et al. [2] also failed to find a significant association between these psychobiological parameters and psychological response to acute stress. This could be explained by the employment of general scores for psychological state variables instead of specific subscales (e.g., fatigue, vigor, depression…), but we prefer to analyse general scores in order to increase power for effect size. Hence, our findings are in line with previous results in this field of research.

The current study is not without limitations. Causality cannot be inferred, as it is a cross-sectional research. Even though the design is strong, it could be argued that the sample size of each group weakens the results. Moreover, our study featured many measures of Csal and IgA during the stress protocol, as well as a control group, which was matched with cases for the main demographic characteristics. Nevertheless, due to the importance of our preliminary findings, a more exhaustive measurement of immune responses is needed. Moreover, heavy nicotine consumption during long-term periods tends to disrupt HPA functioning [23]. Hence, this should be considered in future research. The interpretation of potential interactions in the immune–endocrine network is limited by the lack of data on pro-inflammatory cytokine levels. Future studies should attempt to replicate the results of this study with other samples of informal caregivers, as this could add to the body of evidence to support the idea of dysregulation in both hormonal and immune responses to stress in chronically stressed populations.

## 6. Conclusions

These findings advance our understanding of how a chronically stressed population reacts to acute stress. Dysregulated stress-induced endocrine and immune responses could directly affect the adaptive stress response of caregivers and alter their ability to cope with every day challenges. The physiological ability of caregivers to cope with stressors might be compromised by the aforementioned reasons, regardless of the care context. Developing and implementing psychotherapeutic interventions focused on stress management, as previously done for caregivers of people with ASD [24,25], can be expected help caregivers to reduce their stress levels and cope effectively with stressors. Further studies exploring any potential changes in the endocrine and immune response in caregivers as a result of a psychotherapeutic intervention should be conducted, focusing on whether this type of interventions are helpful for restoring of effective stress management in caregivers of people with EDs.

## Figures and Tables

**Table 1:** Mean ± SD of age and body mass index and demographic variables for caregivers and non-caregivers and care recipient characteristics. ** *p* < 0.001 * *p* < 0.05.

Demographic Variables		ED Caregivers (*n* = 29)	Non-Caregivers (*n* = 36)
Gender			
Male		34%	44%
Female		66%	56%
Age, years **		51.34 ± 4.81	47.07 ± 3.58
Body mass index, kg/m^2^		25.95 ± 3.98	26.40 ± 4.45
Number of children		2.45 ± 1.94	1.89 ± 0.82
Number of children at home		2.10 ± 1.70	1.86 ± 0.80
Menstrual status *	Luteal phase (1–14)	52%	20%
	Follicular phase (15-menstrual period)	16%	45%
	Amenorrhea (>6 months)	32%	35%
	<12 years	17%	6%
Educational level	College degree or higher	83%	94%
Marital status	Married/Cohabiting	86%	92%
	Divorced/Widowed/Single	14%	8%
Employment status	Employed	79%	77%
	Unemployed	18%	20%
	Retired/other	3%	3%
Care recipient characteristics			
Age of care recipient		21.86 ± 5.34	
Diagnosis of the care recipient Anorexia Nervosa		72%	
Bulimia Nervosa		28%	
Gender of care recipient			
Male		7%	
Female		93%	
Caring years from definitive diagnosis		5.53 ± 3.69	
Weekly time caregiving (hours)		35.69 ± 32.00	
Independence (Barthel index)		95.39 ± 19.64	
Disability rating, %		1.13 ± 6.13	

ED, eating disorder.
